# β_1_- and β_2_-adrenergic stimulation-induced electrogenic transport by human endolymphatic sac epithelium and its clinical implications

**DOI:** 10.1038/srep42217

**Published:** 2017-02-06

**Authors:** Bo Gyung Kim, Jin Young Kim, JinSei Jung, In Seok Moon, Joo-Heon Yoon, Jae Young Choi, Sung Huhn Kim

**Affiliations:** 1 Department of Otorhinolaryngology, Soonchunhyang University College of Medicine, Bucheon, 420-767, Republic of Korea; 2 Research Center for Natural Human Defense System, Brain Korea 21 PLUS Project for Medical Science, Yonsei University College of Medicine, Republic of Korea; 3 Department of Otorhinolaryngology, Yonsei University College of Medicine; 4 The Airway Mucus Institute, Yonsei University College of Medicine, Seoul, 03722, Republic of Korea

## Abstract

The endolymphatic sac (ES) is a cystic structure of the inner ear connected to the cochlea and vestibule, which plays a role in regulating ion homeostasis in inner ear fluid. Disruption of ion homeostasis can cause inner ear disorders with hearing loss and dizziness, such as Meniere’s disease. Herein, we found, for the first time, functional evidence for the involvement of β_1_- and β_2_-adrenergic receptors in apical electrogenic ion transport by human ES epithelium by using electrophysiological/pharmacological and molecular biological methods, which were dependent on K^+^ and Cl^−^ ion transport. The apical electrogenic transport was absent or very weak in ES epithelia of patients with Meniere’s disease. These results suggested that adrenergic stimulation via β_1_- and β_2_-adrenergic receptors in the human ES was involved in regulation of inner ear fluid ion homeostasis and impairment of this response could be a pathological mechanism of Meniere’s disease.

The inner ear is composed of three main structures: the cochlea, vestibule, and endolymphatic sac (ES) (Fig. 1). The inner ear structures form luminal structures lined with epithelial cells and connected through narrow lumen. The cochlea and vestibule are peripheral sensory organs that detect sound and angular/linear acceleration, respectively; however, ES is a simple cystic structure and does not contribute to detection of these stimuli. Each structure of the inner ear is filled with fluid of a unique ion composition called the endolymph, which is essential for maintaining hearing and balance. Endolymph in the cochlea and vestibule has high [K^+^] (~150 mM) and low [Na^+^] (~1.5–9 mM), whereas that in the ES has high [Na^+^] (~129 mM) and low [K^+^] (~8–13 mM) (Fig. 1)^1^. High [K^+^] in the cochlea and vestibule enables depolarization of the sensory epithelium by providing K^+^ions to the cytoplasm efficiently through mechano-sensitive non-selective cation channels, which propagate sound and angular/linear acceleration stimulation transmission to the central nervous system through the vestibulocochlear nerve^2^,^3^. The ES is believed to be involved in the regulation of ion homeostasis and endolymph volume^4^; however, the role of high [Na^+^] and low [K^+^] in the luminal fluid, as well as the regulatory mechanisms for ion homeostasis and endolymph volume, have not been definitively proved.

It has been reported that various molecular and physiological mechanisms for the stimulation of endocrine and paracrine secretory molecules are involved in the regulation of inner ear ion homeostasis^5^,^6^,^7^,^8^. Adrenergic stimulation-induced ion transport is believed to be one of the mechanisms of inner ear fluid ion homeostasis. The stimulation acts through adrenergic receptors which are distributed widely in the inner epithelial cells^9^,^10^. Although both α and β-adrenergic receptors are expressed in the inner ear epithelium, relevant functional evidence for the involvement of adrenergic receptors in the regulation of ion homeostasis has mostly been found in β_1_- and β_2_-adrenergic receptors^7^,^11^. These receptors were also reported to exist in the luminal epithelium of the ES^12^. They are believed to play an important role in the regulation of hydrostatic pressure and the ES potential that could be generated by the various ion transports in the ES epithelium. However, the functional evidence for the role of these receptors in inner ear fluid homeostasis and fluid volume regulation has only been provided by animal experiments^7^,^13^,^14^,^15^,^16^ and there have been no reports demonstrating the functional role of the receptors in the human inner ear.

These adrenergic stimulation-induced changes in ion transport in the inner ear are likely to be involved in the pathophysiology of Meniere’s disease, which is an inner ear disorder characterized by recurrent vertigo attacks, sudden hearing loss, tinnitus, and aural fullness. Although the pathological mechanism of the disorder has not been clearly identified, it is believed that the main pathological finding is endolymphatic hydrops, which is a phenomenon of excessive accumulation of endolymph in the inner ear. Endolymphatic hydrops is assumed to be caused by impairment of inner ear fluid volume regulation, of which the main cause has been suggested to be deterioration of ion transport as the movement of major ions in the fluid induces water movement^17^,^18^. The deterioration of ion homeostasis and endolymphatic hydrops can disrupt the normal function of inner ear sensory epithelial cells and their neighbouring extra-sensory epithelium, consequently causing loss of hearing and balance. One of the main triggers for the aggravation of Meniere’s disease symptoms is physical and emotional stress^19^; therefore, it has been suggested that adrenergic stimulation-mediated regulation of inner ear ion transport is involved in the pathophysiology of the disorder^7^,^20^. In this aspect, the roles of ES and β_1_- and β_2_-adrenergic receptors in the luminal epithelium of the ES could be important to the pathophysiology of Meniere’s disease. However, there has been no functional studies for the role of the receptors in the human ES, as described above; furthermore, the functional evidence for the pathological mechanism in the human ES in Meniere’s disease has not been reported elsewhere. Functional evidence from human samples is important for elucidating the pathological mechanism of Meniere’s disease because there are no definite animal models reflecting the condition of human Meniere’s disease. So far, there has been only one functional study about the electrogenic transport in human endolymphatic sac epithelium in which the functional evidence of K^+^ ion channel-driven electrogenic transport in the human ES was identified and it provided an important insight into ion homeostasis of the inner ear^1^. In this study, we attempted to investigate the role of β_1_- and β_2_- adrenergic stimulation in the electrogenic transport of the human ES epithelium and to investigate the difference in adrenergic stimulation-induced electrogenic transport between the ES of patients with Meniere’s disease and disease-free controls, using electrophysiological, pharmacological, and molecular biological methods. This study can provide the first functional evidence for the role of β-adrenergic receptors in the ion homeostasis of the human inner ear and insight into a basis for the pathophysiology of Meniere’s disease.

## Results

### Transcript expression of β_1_- and β_2_-adrenergic receptors in human ES

**Figure 2:**
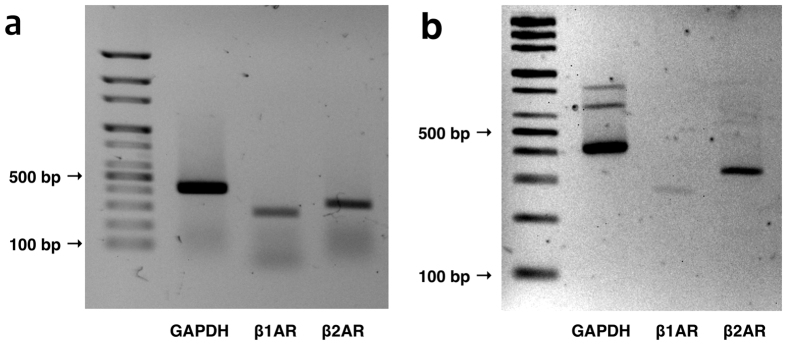
Full length gel images of transcript expression of β_1_- and β_2_-adrenergic receptors. (**a**) Receptor transcript expression in a positive control (human heart). (**b**) Receptor transcript expression in the human endolymphatic sac.

Before investigating functional evidence for β_1_- and β_2_-adrenergic receptors, we investigated whether there were transcripts of these receptors in normal human ES tissue. RT-PCR revealed product bands of relevant base pairs for β_1_- and β_2_-adrenergic receptors in the human ES (Fig. 2).

### β_1_- and β_2_-adrenergic receptor-mediated electrogenic transport by theES epithelium of disease-free controls

**Figure 3:**
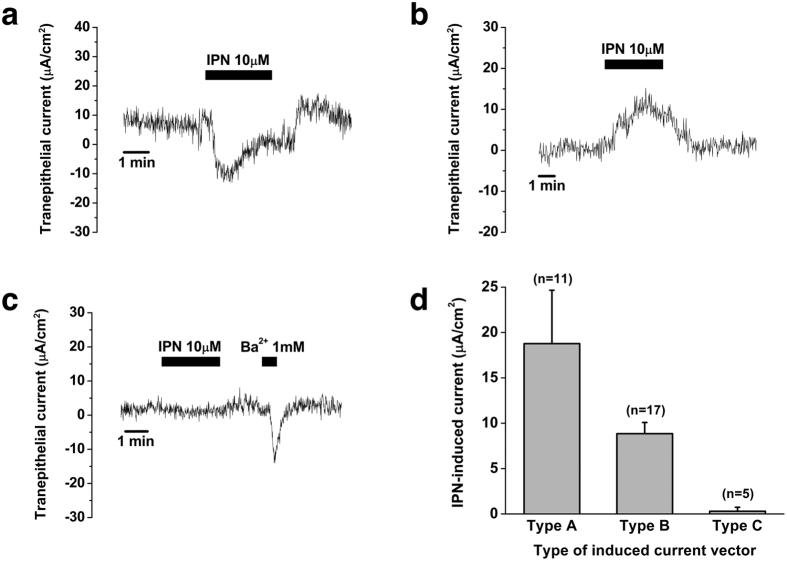
Isoproterenol (10 μM)-induced electrogenic transport by the human endolymphatic sac epithelium. (**a**) Representative figure of isoproterenol-induced cation absorption/anion secretion current (type A). (**b**) Representative figure of isoproterenol-induced cation secretion/anion absorption current (type B). (**c**) Representative figure showing no effect of isoproterenol in the electrogenic transport (type C). Tissue viability was assessed by the detection of the current change after Ba^2+^ (1 mM) application. (**d**) Mean amount of current change induced by isoproterenol in type A, type B, and type C. IPN, isoproterenol.

Isoproterenol (10 μM), a nonspecific agonist for β_1_- and β_2_-adrenergic receptors, was used to investigate the effect of β-adrenergic stimulation on electrogenic transport in the human ES epithelium. Isoproterenol induced three different types of trans-epithelial current vectors: after the perfusion of isoproterenol, the apical trans-epithelial current vector of cation absorption/anion secretion (type A) and cation secretion/anion absorption (type B) was induced in 11 samples and 17 samples, respectively. However, no current change was observed in 5 samples (type C) (Fig. 3a–c). The mean current changes in type A and type B currents induced by isoproterenol were 18.8 ± 5.9 μA/cm^2^ (from 1.7 ± 1.3 to –17.1 ± 6.6 μA/cm^2^, n = 11) and 8.8 ± 1.2 μA/cm^2^ (from 2.1 ± 2.1 to 10.9 ± 2.5 μA/cm^2^, n = 17), respectively (Fig. 3d). There was no significant difference in the amount of isoproterenol-induced trans-epithelial current between types A and B (p > 0.05). The type of isoproterenol-induced current vectors was different according to the location of measurement (proximal, middle, and distal portion of the ES), but the types of current were not consistent with the location of measurement.

**Figure 4:**
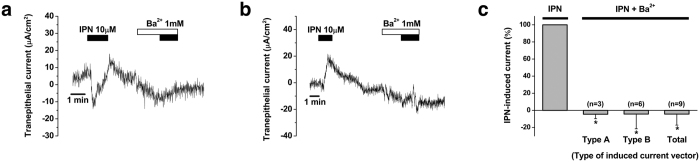
Inhibition of isoproterenol-induced electrogenic transport by Ba^2+^ (1 mM) in human endolymphatic sac epithelium. (**a**) Representative figure showing the effect of Ba^2+^ in type A current. (**b**) Representative figure showing the effect of Ba^2+^ in type B current. (**c**) Mean inhibitory effect of Ba^2+^ for isoproterenol-induced trans-epithelial current. IPN, isoproterenol. *p < 0.05.

We investigated whether isoproterenol-induced electrogenic transport was dependent on K^+^ channels using Ba^2+^ (1 mM), a non-specific K^+^ channel blocker, because K^+^ channel-dependent electrogenic transport was only functionally identified in the human ES^1^. We investigated the inhibitory effect of Ba^2+^ on human ES tissue in which the type A and B trans-epithelial currents induced by isoproterenol were detected. Before application of Ba^2+^, isoproterenol induced type A (n = 3) and type B (n = 6) trans-epithelial currents, as described above (Fig. 4a and b). When isoproterenol was applied during the perfusion of Ba^2+^, isoproterenol-induced trans-epithelial current was inhibited by 104.1 ± 5.7% (from16.0 ± 2.2 μA/cm^2^ to 0.6 ± 0.7 μA/cm^2^, p < 0.001) for type A trans-epithelial current, 104.3 ± 18.6% (from 12.2 ± 2.7 μA/cm^2^ to 1.7 ± 2.8 μA/cm^2^, p = 0.002) for type B trans-epithelial current, and 104.3 ± 12.9% as a whole (from 13.1 ± 2.0 μA/cm^2^ to 1.1 ± 2.0 μA/cm^2^, p < 0.001) (Fig. 4a–c).

**Figure 5:**
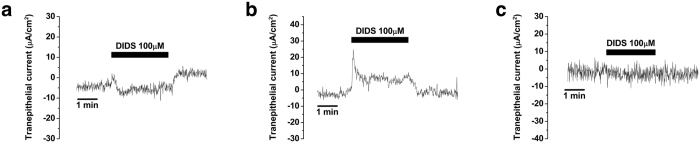
Changes in electrogenic transport induced by DIDS (100 μM) in the human endolymphatic sac epithelium. (**a**) Representative figure of DIDS-induced cation absorption/anion secretion current. (**b**) Representative figure of DIDS-induced cation secretion/anion absorption current. (**c**) Representative figure showing no effect of DIDS on electrogenic transport. DIDS, 4,4′-diisothiocyano-2,2′-stilbenedisulfonic acid.

We investigated whether Cl^−^ channel-dependent electrogenic transport was also involved in β-adrenergic stimulation-induced trans-epithelial currents, because anion transport should be balanced with the transport of cations and Cl^−^ is the most abundant anion in the luminal fluid of the ES. In addition, isoproterenol-induced Cl^−^ transport was already reported in the other epithelial cells of the inner ear^7^. First, 4,4′-diisothiocyano-2,2′-stilbenedisulfonic acid (DIDS, 100 μM), a non-specific blocker of anion transport such as Cl^−^ channels except for cAMP-regulated Cl^−^ channels and anion exchangers, was applied during the measurement of trans-epithelial current from the human ES epithelium to examine whether there was Cl^−^ ion-dependent apical electrogenic transport. Application of DIDS decreased either basal cation absorption/anion secretion or cation secretion/anion absorption by 6.8 ± 1.7 μA/cm^2^ (from −0.1 ± 1.6 μA/cm^2^ to 6.7 ± 0.4 μA/cm^2^, n = 5) and 17.0 ± 8.6 μA/cm^2^ (from −1.3 ± 2.2 μA/cm^2^ to −18.3 ± 8.1 μA/cm^2^, n = 5), respectively (Fig. 5a and b). However, it showed no effects on electrogenic transport in the 3 samples (Fig. 5c).

**Figure 6:**
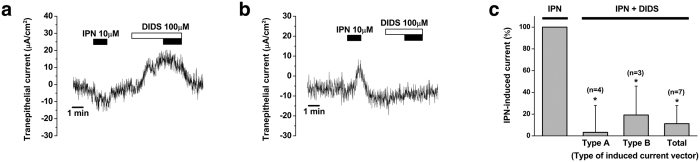
Inhibition of isoproterenol-induced electrogenic transport by DIDS (100 μM) in the human endolymphatic sac epithelium. (**a**) Representative figure showing the effect of DIDS on type A current. (**b**) Representative figure showing the effect of DIDS on type B current. (**c**) Mean inhibitory effect of DIDS for isoproterenol-induced trans-epithelial current. IPN, isoproterenol; DIDS, 4,4′-diisothiocyano-2,2′-stilbenedisulfonic acid; *p < 0.05.

We investigated the effect of DIDS on isoproterenol-induced electrogenic transport by human ES tissues in which type A or type B trans-epithelial current was detected. Application of DIDS inhibited either type A or type B trans-epithelial current by 96.8 ± 24.7% (from −14.3 ± 2.1 μA/cm^2^ to 0.2 ± 2.1 μA/cm^2^, n = 4, p = 0.01) or 80.1 ± 26.6% (from 5.8 ± 1.3 μA/cm^2^ to 0.6 ± 1.7 μA/cm^2^, n = 3, p = 0.04), respectively, and by 88.8 ± 16.6% (from 10.1 ± 2.2 μA/cm^2^ to 0.2 ± 1.6 μA/cm^2^, n = 7, p = 0.002) as a whole (Fig. 6a–c).

**Figure 7:**
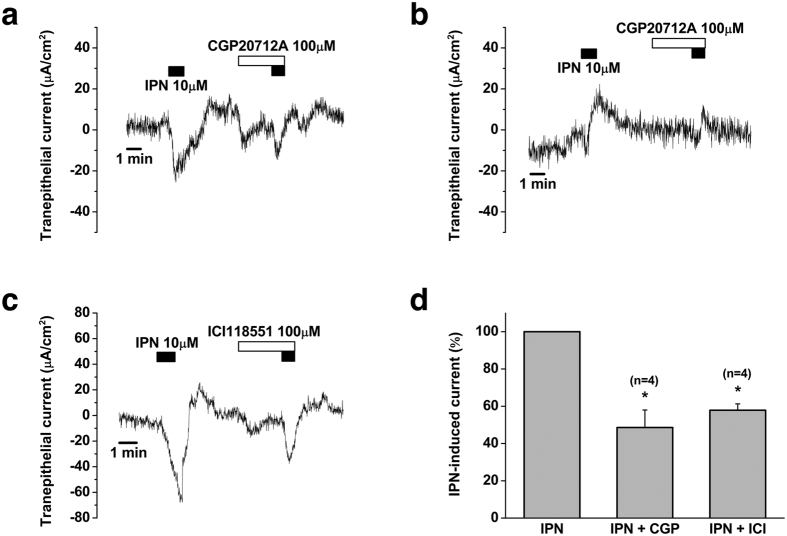
Effect of selective β-adrenergic receptor blockers on isoproterenol-induced electrogenic transport. (**a** and **b**). Representative figures of the inhibitory effects of β_1_-adrenergic receptor blocker (CGP20712A) on type A and B isoproterenol-induced electrogenic transport. (**c**). Representative figure showing the inhibitory effect of β_2_-adrenergic receptor blocker (ICI18551) on type A isoproterenol-induced electrogenic transport. (**d**) Mean inhibitory effects of CGP20712A and ICI18551 on isoproterenol-induced electrogenic transport. *p < 0.05.

Then, we applied selective blockers for each β_1_- and β_2_-adrenergic receptor (100 μM CGP-20712A for β_1_ blocker and 100 μM ICI118551 for β_2_ blocker) to identify which subtype of β-adrenergic receptor was involved in isoproterenol-induced electrogenic transport. CGP-20712A inhibited isoproterenol-induced trans-epithelial current by 55.3 ± 12.0% (from 26.4 ± 18.7 μA/cm^2^ to 15.8 ± 12.6 μA/cm^2^, p = 0.001, n = 4) in which type A current was reduced by 56.4 ± 20.8% (from 33.6 ± 30.0 μA/cm^2^ to 20.9 ± 20.0 μA/cm^2^, n = 3) and type B current by 53.3% (from 12.2 μA/cm^2^ to 5.7 μA/cm^2^, n = 1) (Fig. 7a,b and d). The effect of ICI118551 could be investigated only in type A current because we could not detect type B current during the experiment. ICI118551 inhibited isoproterenol-induced trans-epithelial current by 42.1 ± 3.4% (from 22.4 ± 9.7 μA/cm^2^ to 12.0 ± 5.0 μA/cm^2^, p = 0.042, n = 4) (Fig. 7c and d). Consequently, both CGP-20712A and ICI118551 partially inhibited (~50%) isoproterenol-induced apical electrogenic transport by human ES epithelium (Fig. 7d).

These results indicated that isoproterenol stimulated K^+^ channel-dependent and Cl^−^ channel-dependent apical electrogenic transport, which was mediated by both β_1_- and β_2_-adrenergic receptors in the human ES epithelium.

### β_1_- and β_2_- adrenergic receptor-mediated electrogenic transport in the ES epithelium of patients with Meniere’s disease

**Figure 8:**
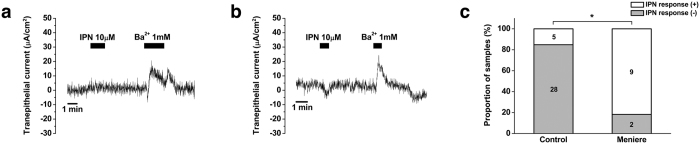
Effect of isoproterenol on electrogenic transport by human endolymphatic sac epithelia inpatients with Meniere’s disease. (**a**) Representative figure showing no effect of isoproterenol on electrogenic transport. (**b**) Representative figure of isoproterenol-induced small type B current. (**c**) Differences in the presence of isoproterenol-induced electrogenic transport between disease-free controls and patients with Meniere’s disease. IPN, isoproterenol; numbers in the bar graph represent the number of samples; *p < 0.05.

Next, we investigated whether there are any differences in β_1_- and β_2_-adrenergic receptor-mediated apical electrogenic transport in the ES of patients with Meniere’s disease. Interestingly, 100 μM isoproterenol did not induce any changes in the trans-epithelial current from the ES epithelium in 81.8% (9/11) of samples with Meniere’s disease (from 1.7 ± 0.8 μA/cm^2^ to 1.1 ± 0.8 μA/cm^2^, p > 0.05, Fig. 8a and c). In two samples (patient 3 and 5 in Supplementary Table S1), type B current was detected after the perfusion of isoproterenol (Fig. 8b); however, the isoproterenol-induced current was much smaller than that of the disease-free controls (4.3 and 2.4 μA/cm^2^ in Meniere’s disease vs. 17.1 ± 6.6 for type A current and 10.9 ± 2.5 μA/cm^2^ for type B current in disease-free controls). After the application of isoproterenol, Ba^2+^ (1 mM) was applied to confirm the viability of the tissue (Fig. 8a), which was expected to decrease the cation section/anion absorption current, as reported previously^1^. Perfusion of Ba^2+^ after the application of isoproterenol demonstrated a decrease in the apical trans-epithelial cation secretion/anion absorption by 19.4 ± 5.9 μA/cm^2^.

### Protein expression of β_1_- and β_2_-adrenergic receptors in the ES epithelium of disease-free controls and patients with Meniere’s disease

**Figure 9:**
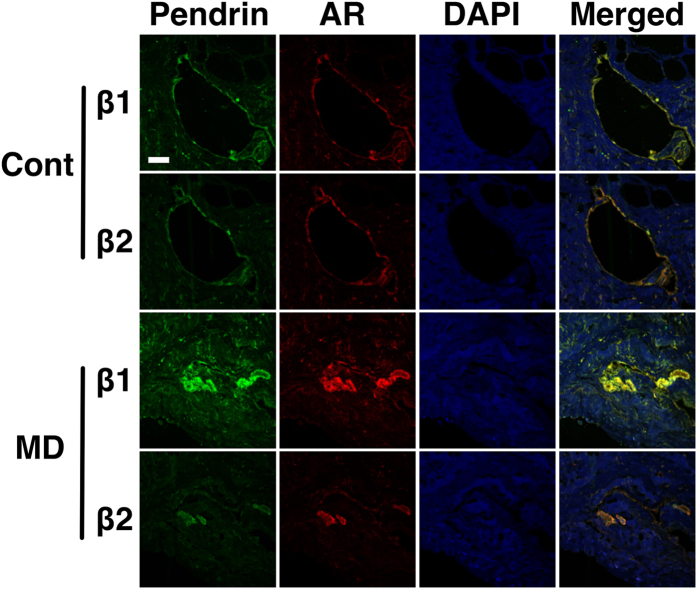
Protein expression of β_1_- and β_2_-adrenergic receptors in the human endolymphatic sac epithelium of disease-free controls and patients with Meniere’s disease. AR, adrenergic receptors; Cont, disease-free controls; MD, Meniere’s disease; β1, β_1_-adrenergic receptor; β2, β_2_-adrenergic receptor. Scale bar in the left uppermost figure indicates 50 μm.

Finally, we investigated whether there were any differences in the protein expression of β_1_- and β_2_-adrenergic receptors in the ES epithelium between disease-free controls and Meniere’s disease using immunofluorescent staining. Both β_1_- and β_2_- adrenergic receptors were revealed to be expressed in the ES epithelium of disease-free controls and patients with Meniere’s disease (Fig. 9). The distribution of the receptors was mostly co-localized with pendrin (Fig. 9), representing the localization of the receptors in mitochondria-rich cells. This result suggested that the impaired response to isoproterenol stimulation in the ES epithelium of patients with Meniere’s disease was not likely to be caused by the depletion of β_1_- and β_2_-adrenergic receptors but rather by the functional impairment of the receptors or the related-ion channels.

## Discussion

This study was the first functional study that demonstrated the presence and role of β_1_- and β_2_- adrenergic receptors in electrogenic transport by the human ES epithelium. Furthermore, we demonstrated the difference in adrenergic stimulation-induced electrogenic transport between disease-free controls and patients with Meniere’s disease, which may suggest the pathological mechanism of Meniere’s disease.

We used a scanning vibrating electrode technique that is suitable for the measurement of current density from small area of epithelium for functional studies for several reasons. First, the system could measure the whole trans-epithelial current, which could be more physiologically relevant compared to current measurements in the single cell, because water movement usually follows changes in whole ion movement, which can induce changes in the osmotic gradient. Second, the human ES epithelium consists of five different cell types, in which the distribution of ion channels and their roles are believed to be different^21^. It is impossible to accurately identify epithelial cell types in the human ES during experiments using patch clamps for the measurement of current from a single cell. Therefore, we believe that the scanning vibrating electrode technique was appropriate for the purposes of our study.

Adrenergic stimulation is believed to be involved in the regulation of microvascular tone, ion homeostasis, endolymphatic pressure, and autonomic nervous system function in the inner ear^11^,^15^,^22^,^23^. Various α- and β- adrenergic receptors have been proven to be distributed in the epithelial cells, vessels, and ganglions in the whole inner ear organs, and the receptors in the epithelial cells of the inner ear are assumed to be involved mainly in the regulation of ion transport activities. So far, very few functional studies of the role of the adrenergic receptors in ion homeostasis in the inner ear have been reported. In the cochlear and vestibular organs, it was identified that β_1_-adrenergic receptors stimulated K^+^ secretion in vestibular dark cells and strial marginal cells, and β_2_-adrenergic receptors stimulated Cl^−^ secretion in the semicircular canal epithelial cells^7^,^11^. In the ES, β-adrenergic stimulation using isoproterenol caused increased cochlear hydrostatic pressure and decreased ES potential, which were not induced when the ES was obstructed^15^. This study indicated that the ES was likely to be involved in the regulation of hydrostatic pressure in the cochlea by β-adrenergic receptors, although the subtypes of the receptor and the mechanism of regulation were not directly identified. In our study, application of isoproterenol induced three different types of current vectors (type A for cation absorption/anion secretion, type B for cation secretion/anion absorption, and type C for no response to isoproterenol) in ES epithelium. This outcome may have resulted from various histological epithelial cell types of the ES, and the type of current vector could be dependent on the position of current measurement, which was also reported regarding the basal current in the human ES^1^. Regardless of the type of induced-current vector, isoproterenol-induced electrogenic transport was fully inhibited by Ba^2+^ and DIDS. These findings suggested that both K^+^ and Cl^−^ ion channels were involved in the generation of apical electrogenic transport by the stimulation of β-adrenergic receptors, although the location (apical or basolateral) and definite types of the channels are uncertain. These channels can exist on the apical, basolateral, or both surfaces of ES epithelial cells, and they can induce isoproterenol-induced electrogenic transport though the channel itself and/or can be involved in the transport of ions in other channels on the apical surface by regulation of K^+^ and Cl^−^ ion transport. The latter hypothesis is more persuasive in that both Ba^2+^ and DIDS fully inhibited isoproterenol-induced electrogenic transport; if these channels were located on the apical surface and induced electrogenic transport through the channel itself by the stimulation of isoproterenol, Ba^2+^ and DIDS partially inhibited this current, depending on the density of the ion channels on the apical surface. Although this is less likely to occur when considering the heterogeneity of cell types in the small area of the ES epithelium, these K^+^ and Cl^−^ ion channels could be separately located on apical surfaces of certain cell types and the blockers could inhibit the whole apical cation secretion (K^+^ channels) or anion secretion (Cl^−^ channels). Additionally, the effect of DIDS reflects the involvement of Cl^−^ channels in the generation of baseline apical electrogenic transport in the same manner. Application of DIDS induced three different apical trans-epithelial current vector changes from the baseline trans-epithelial current (inhibition of apical cation absorption/anion secretion, cation secretion/anion absorption or no response), suggesting that Cl^−^ ion channels involved in the generation of various apical electrogenic transport by affecting the apical ion channel activity through apical and/or basolateral Cl^−^ ion transport or that apical Cl^−^ ion channel itself could be the main target for DIDS in certain cell types. The K^+^ channels involved in the generation of isoproterenol-induced electrogenic transport could be KCNJ14, KCNK2, and KCNK6, which were previously identified in functional studies, and their activities were inhibited by 1 mM Ba^2+^ in human ES epithelium^1^.

We identified isoproterenol-induced electrogenic transport via β_1_- or β_2_- adrenergic receptor by applying selective β_1_- and β_2_-adrenergic receptor inhibitors (CGP20712A and ICI18551). In addition to the presence of β_1_- and β_2_-adrenergic receptor, the β_3_-adrenergic receptor, which was not found in the stria vascularis of the cochlea, was revealed to exist in the intermediate portion of the rat ES epithelium, recently^12^. The β_3_-adrenergic receptor showed the strongest expression in immunohistochemistry compared to the β_1_- and β_2_- adrenergic receptors, and the authors suggested that the β_3_-adrenergic receptor might play an important role in the ES. We used the extraosseous portion of the human ES, which could have different distributions of ion channels and their regulators from the intermediate portion (intraosseous portion), and we cannot be sure whether there was any activity of β_3_-adrenergic receptor in the extraosseous portion of the human ES. We did not investigate the functional role of the β_3_-adrenergic receptor because of the limitation of human samples for this experiment and because there have not yet been any reports of a functional role of β_3_-adrenergic receptor in the inner ear. Opportunities to harvest human ES samples were only possible during function-destructive surgery, such as acoustic tumour removal via the translabyrinthine approach. Furthermore, opportunities for harvesting the ES in patients with Meniere’s disease were limited because of the small number of patients who undergo ES surgery owing to the recent development of less invasive methods in managing intractable Meniere’s disease such as intratympanic gentamicin and steroid. Therefore, we focused on the investigation of β_1_- and β_2_- adrenergic receptor activity, of which functional studies were previously reported in the inner ear for the efficient use of tissue samples. However, the expression and function of β_3_-adrenergic receptors in human ES should be investigated in future studies, which could provide evidence for elucidating the role of adrenergic stimulation in ion homeostasis in the inner ear.

The most interesting finding in the adrenergic stimulation-induced activity between disease-free controls and patients with Meniere’s disease was the absence or very weak presence of isoproterenol-induced electrogenic transport in the ES of patients with Meniere’s disease, which was rare in the disease-free controls. The response to adrenergic stimulation in ES can occur in conjunction with other adrenergic receptor activity in the cochlea and vestibule, such as stria vascularis, dark cells, and the semicircular canal epithelium. If one of the responses to adrenergic stimulation in each inner ear organ was impaired, the ion balance in the inner ear could be disturbed and could finally cause disruption of inner ear function, such as hearing impairment and vertigo. The possible involvement of autonomic nervous system in Meniere’s disease has been previously reported^24^,^25^, and the findings of our study suggested that the impaired response to adrenergic stimulation could be one of the pathological mechanisms of the disease. The weak or impaired response to adrenergic stimulation in the ES of patients with Meniere’s disease is likely to be caused by a defect in downstream pathways in adrenergic receptor signalling or the dysfunction of ion channels involved in the adrenergic stimulation-induced electrogenic transport because the expression of β_1_- and β_2_-adrenergic receptors was also strongly detected in ES epithelia of patients with Meniere’s disease. Although the fundamental cause for the deterioration of the impaired response was not clear, it is tempting to speculate that various etiologies, such as viral, autoimmune or inflammatory response, allergy, trauma, and genetic causes, suggested by previous literatures could insult endolymphatic sac epithelium and caused deterioration in the downstream pathways of β-adrenergic stimulation-induced response and/or dysfunction of ion channels involved in the response to β_1_- and β_2_-adrenergic stimulation. Adrenergic stimulation via β_1_- and β_2_- adrenergic receptors is transmitted via G-protein-coupled adenylyl cyclase and protein kinase A-dependent pathway, and the receptor function is also modulated by the phosphorylation of β_2_-adrenergic receptor by protein kinase A and C (“feed back” and “cross system” phosphorylation)^26^,^27^. Recently, it was reported that a missense variant of PRKCB gene that encoding a subunit of protein kinase C was identified in a family with Meniere’s disease^28^, which supports that the deterioration of adrenergic receptor signaling pathway could be one of possible pathologic mechanism of Meniere’s disease. Alternatively, adrenergic signalling may not impaired but there may be a functional deficit in K^+^, Cl^−^, or other ion channels involved in the adrenergic stimulation-induced electrogenic transport. Future studies of β-adrenergic receptor signalling pathways (measurement of differences in the levels of cAMP and molecular analysis of PKA and PKC pathway for example) and difference in the expression and function of ion channels responsive to adrenergic stimulation in patients with Meniere’s disease could provide evidence for the pathological mechanism of Meniere’s disease. Nevertheless, further studies are required to get a sufficient number of ES samples to obtain reliable results.

In conclusion, adrenergic stimulation induced apical electrogenic transport in the human ES epithelium via β_1_- and β_2_-adrenergic receptors, and electrogenic transport was dependent on K^+^ and Cl^−^ ion channel activities. Adrenergic stimulation-induced electrogenic transport activity was impaired in patients with Meniere’s disease, which could be a pathological mechanism of Meniere’s disease.

## Methods

### Selection of patients

Nineteen patients with acoustic schwannoma for disease-free controls and 10 patients with definite Meniere’s disease, as diagnosed using the 1995 diagnostic criteria of the American Academy of Otolaryngology-Head and Neck Surgery, were enrolled in this study. These patients also could be diagnosed as definite Meniere’s disease according to the newly established diagnostic criteria of Barany Society^29^. Patients with acoustic schwannoma (M:F = 6:13; mean age: 52.5 ± 3.1 years) underwent surgical tumour removal via the translabyrinthine approach. Patients with Meniere’s disease (M:F = 5:5; mean age: 51.1 ± 4.3 years) underwent ES surgery because their recurrent vertigo attacks could not be controlled by non- or minimally invasive treatment such as lifestyle modification (low salt diet, avoiding physical and emotional stress), medication (betahistine and diuretics), or intratympanic dexamethasone for more than 3 months. Among the patients, two patients (patient 7 and 10 in Supplementary Table S1) had bilateral Meniere’s disease and one of them (patient 10 in Supplementary Table S1) had vestibular migraine simultaneously. These patients had been treated with endolymphatic sac surgery at first, but it failed to control vertigo attacks. Consequently, they underwent chemical labyrinthectomy using streptomycin, which resulted profound hearing loss in the treated ears, although vertigo attacks were controlled. Meniere’s disease in the other side developed at 7 and 8 years after the onset of first Meniere’s disease in those patients, respectively, and they underwent ES surgery again in the recurred side. Those patients finally underwent cochlear implantation due to the bilateral severe to profound hearing loss. Patient 5 (Supplementary Table S1) also underwent chemical labyrinthectomy 4 times due to the failure in controlling vertigo with ES surgery and finally had profound hearing loss on the lesion side. Patient 8 (Supplementary Table S1) had familial Meniere’s disease with autosomal dominant inheritance pattern. None of the patients had a history of systemic autoimmune disorder.

Information about the patients’ demographics, hearing threshold (average of hearing threshold at 500, 1000, 2000, and 4000 Hz), vestibular asymmetry measured by Jonkee’s formula in bithermal caloric tests^30^,^31^, and mean number of vertigo attacks/month over the 3 months before surgery, family history of Meniere’s disease, and, comorbidity of vestibular migraine^31^ which showed higher incidence in Meniere’s disease than that in normal population is described in Supplementary Table S1.

### Ethics statement

This study was approved by the institutional review board of Severance Hospital, Yonsei University College of Medicine (approval number: 4-2013-0483) and written informed consent was obtained from each of the participants. The experimental methods were performed in accordance with the approved guidelines.

### Harvesting ES

For disease-free controls, the ES was harvested using the same method as previously described during acoustic tumour removal via the translabyrinthine approach^1^. Briefly, the ES was exposed during the surgical procedure, and the whole ES was harvested after inflation of the ES by the injection of ~200 μl of normal saline to harvest the tissue without injury to the luminal surface during dissection. For patients with Meniere’s disease, ES surgery was performed. After complete mastoidectomy, the bony covering of the posterior cranial fossa was carefully removed, and the ES and endolymphatic duct were exposed. After exposure, only a small area (~3 × 3 to 4 × 4 mm) of the cystic portion was harvested to minimize hearing deterioration after surgery after the injection of ~200 μl of normal saline, and then the luminal portion of the ES was dissected through the defect of the cystic portion from which the tissue was harvested, and a T-shaped silastic sheet was inserted into the lumen to secure the shunt of the ES. The harvested ES was immersed in perilymph-like solution (specified in the section, *Measurement of trans-epithelial current from ES epithelium*) and was immediately used for the experiment.

For electrophysiological experiment, harvested ES was prepared in small size (~2 × 2 to 3 × 3 mm) before mounting in the stage of microscope. Therefore, multiple segments of tissue from one patient were used for each measurement in disease-free control; however, only a small ES segment was harvested from the patients with Meniere’s disease, one tissue from one patient was used for each measurement except in patient 2 (Supplementary Table S1); tissue from patient 2 was divided into two segments and used for each measurement. For immunofluorescent staining, we prepared a small piece (~3 × 3 mm) from the harvested ES of disease-control (n = 2) and patients with Meniere’s disease (from patient 8 and 9 in Table S1) and immersed the tissue into 4% paraformaldehyde, and the other parts of the tissue from those patients were used for the electrophysiological experiments.

### RT-PCR

We investigated the presence of mRNA for subtypes of the β_1_- and β_2_-adrenergic receptor using disease-free control tissue by RT-PCR. After homogenization of the harvested human ES tissue, total RNA was extracted using TRIzol^®^ (Invitrogen, Carlsbad, CA, USA) following the manufacturer’s protocol. The quantity and quality of isolated RNA were determined with a NanoDrop ND-100 spectrophotometer (NanoDrop Technologies, Wilmington, DE, USA) and by analysing the 18 S and 28 S rRNA bands after electrophoresis. cDNA was synthesized from 3 μg of total RNA with random hexamer primers (Perkin Elmer Life Sciences, Boston, MA, USA; and Roche Applied Science, Mannheim, Germany), AMV reverse transcriptase (Perkin Elmer Life Sciences), and RNase inhibitor (Perkin Elmer Life Sciences). The reverse transcription step was performed for 10 min at room temperature, 30 min at 50 °C, and 15 min at 95 °C. The transcript of each adrenergic receptor was amplified using gene-specific primers (Table 1). The PCR conditions included 30 cycles of denaturation at 94 °C for 30 s, annealing at 54 °C for 30 s, and polymerization at 72 °C for 30 s. The PCR products were run on a 1.5% agarose gel and visualized with ethidium bromide under a transilluminator. Total RNA extracted from the human heart (Clontech, Takara Korea Biomedical Inc., Seoul, South Korea) was used as a positive control for the adrenergic receptors. All PCRs of the products were purified by a PCR purification kit (Qiagen, Valencia, CA, USA), and the resulting purified PCR products were sequenced to verify the identity of the RT-PCR products.

### Measurement of trans-epithelial current from ES epithelium

To measure the trans-epithelial current from the ES epithelium, the scanning vibrating electrode technique was used as previously described^1^. Briefly, the small prepared tissue was folded smoothly with the luminal side out. The tissue was mounted in a perfusion chamber of an inverted microscope (IX51, Olympus, Tokyo, Japan) and continuously perfused at 37 °C with perilymph-like solution with an exchange rate of 1.2 times/s. The current density was monitored by vibrating a platinum-iridium wire microelectrode insulated with parlene-C (Micro Electrodes, Gaithersburg, MD, USA) and coated with Pt black on the exposed tip. The electrode tip of the probe was vibrated at two frequencies between 400 and 700 Hz along the horizontal (*x*) and vertical (*z*) axes by piezo-electric bimorph elements (Applicable Electronics, Forestdale, MA, USA) and was positioned 5 ± 2 μm from the apical surface of the epithelium. The *x*-axis was perpendicular to the face of the epithelium. A platinum-black electrode served as a reference in the bath chamber. The signals from the oscillators driving the probe, which were connected to a dual-channel phase-sensitive detector (Applicable Electronics), were digitized (16 bit) at a rate of 0.5 Hz. The electrode was positioned so that the current density showed a maximum *x* value and a minimum *z* value. The data that were derived from the *x* direction current density were plotted with Origin software, version 8.0. (OriginLab Software, Northampton, MA, USA).

For the investigation of β-adrenergic stimulation-induced current, isoproterenol (10 μM), a nonspecific agonist for β_1_- and β_2_-adrenergic receptors, was applied. Then, we applied Ba^2+^ (1 mM), a non-specific K^+^ channel blocker, before starting the perfusion of isoproterenol and during the perfusion of isoproterenol to investigate if the isoproterenol-induced electrogenic transport was dependent upon Ba^2+^ sensitive K^+^ channels, because the K^+^ channels were already identified in human ES epithelium by functional study^1^. For the next step, the presence of chloride channel and anion exchangers-dependent electrogenic transport was investigated by applying DIDS (100 μM), because Cl^−^ ion is one of the abundant ions in the luminal fluid of ES and usually cation transport is balanced with the transport of anion transport. After that, DIDS (100 μM) was applied before and during the perfusion of isoproterenol to investigate if the isoproterenol-induced electrogenic transport was also dependent upon the DIDS sensitive Cl^−^ channels and anion exchangers. The contribution of each β_1_- and β_2_-adrenergic receptor to the electrogenic transport was investigated by applying each selective blocker for β_1_- and β_2_-adrenergic receptor (100 μM CGP-20712A for β_1_-adrenergic receptor and 100 μM ICI118551 for β_2_-adrenergic receptor). Then the difference of β_1_- and β_2_-adrenergic receptors-dependent electrogenic transport by ES epithelium between disease control and Meniere’s disease were investigated by comparing the presence and the amount of isoproterenol-induced electrogenic transport. In the samples which have not showed any response to isoproterenol, Ba^2+^ (1 mM) was applied to confirm the viability of the tissue. For electrophysiological experiments, a perilymph-like physiological saline solution (150 mMNaCl, 3.6 mMKCl, 1 mM MgCl_2_, 0.7 mM CaCl_2_, 5 mM glucose, and 10 mM HEPES [pH 7.4]) was used for perfusion. The pharmacological agents that were used in this study were purchased from Sigma (St. Louis, MO, USA). All agents were dissolved in the perilymph-like solution before application.

### Immunofluorescent staining

To verify the protein expression of β_1_- and β_2_-adrenergic receptors, immunofluorescent staining of the ES of disease-free control and Meniere’s disease tissues was performed. We used pendrin as a marker for the mitochondria-rich cells of the ES. Human ES tissues were fixed with 4% paraformaldehyde for 24 h and then were dehydrated and embedded in paraffin. Paraffin blocks were sectioned into 5 μm-thick slices and were attached to glass slides. The samples were deparaffinised twice in xylene for 5 min and hydrated with 100% and 95% ethanol twice for 3 min and 2 min, respectively. After washing the sample with distilled water, antigen retrieval was performed in pH 9.0 Tris-EDTA buffer for 20 min, using a microwave. The samples were blocked with blocking solution (Lab Vision Ultra V Block, Thermo Scientific, Waltham, MA, USA) for 1 h and then were washed for 5 min three times in PBS. The samples were incubated overnight at 4 °C with the first primary antibodies (β1-adrenergic receptor [1:100, ab3442, Abcam, Cambridge, MA, USA], β2-adrenergic receptor [1:20, ab135641, Abcam]). After incubation, samples were washed and incubated with secondary antibodies according to the use of the first primary antibodies (1:200 goat anti-rabbit Alexa Fluor 568 [ab175471, Abcam]) for 1 hour at room temperature. The samples were washed and blocked with blocking solution for 1 h. Then, the samples were incubated overnight at 4 °C with the second primary antibody (pendrin, 1:50, sc-23779, Santa Cruz). After incubation, the samples were washed and incubated with secondary antibodies for pendrin (1:200 donkey anti-goat Alexa 488 Fluor [ab150129, Abcam]) for 1 hour at room temperature. The samples were washed, and the nuclei of the tissues were stained with 4′,6-diamidino-2-phenylindole (DAPI). The samples on the glass slides were mounted with cover slips, and the expression levels of the channels were observed using confocal laser scanning microscopy (LSM780, Carl Zeiss, Jena, Germany).

### Data analysis

Results are presented as the means ± SE from n observations. The amount of the trans-epithelial current change was calculated as follows. The basal current amount was calculated by subtracting the current at the reference point (400 μM from the epithelium) from the trans-epithelial current obtained during the first 30 s after locating the probe on the apical side of the epithelium. The changes in current that were induced by the application of the first pharmacological agent were calculated by subtracting the basal current from the trans-epithelial current obtained during the last 30 s after the application of the agent. If the other pharmacological agents were serially applied after the first application of the agent, we waited until the trans-epithelial current was stable after cessation of the first agent, and then applied the next agent. The current changes were calculated by subtracting the trans-epithelial current obtained at 30 s before application of the next pharmacological agent from the trans-epithelial current obtained during the last 30 s of the application of the next agent. The significance of the changes in the current density after the application of pharmacological agents was calculated using Student’s *t*-test or the Mann-Whitney U test. The proportional difference in the response to isoproterenol between disease-free controls and patients with Meniere’s disease was calculated using Fisher’s exact test. A value of p < 0.05 was considered to indicate a statistically significant result.

## Additional Information

**How to cite this article**: Kim, B. G. *et al*. β_1_- and β_2_-adrenergic stimulation-induced electrogenic transport by human endolymphatic sac epithelium and its clinical implications. *Sci. Rep.* **7**, 42217; doi: 10.1038/srep42217 (2017).

**Publisher's note:** Springer Nature remains neutral with regard to jurisdictional claims in published maps and institutional affiliations.

## Supplementary Material

Supplementary Table S1

## Figures and Tables

**Figure 1:**
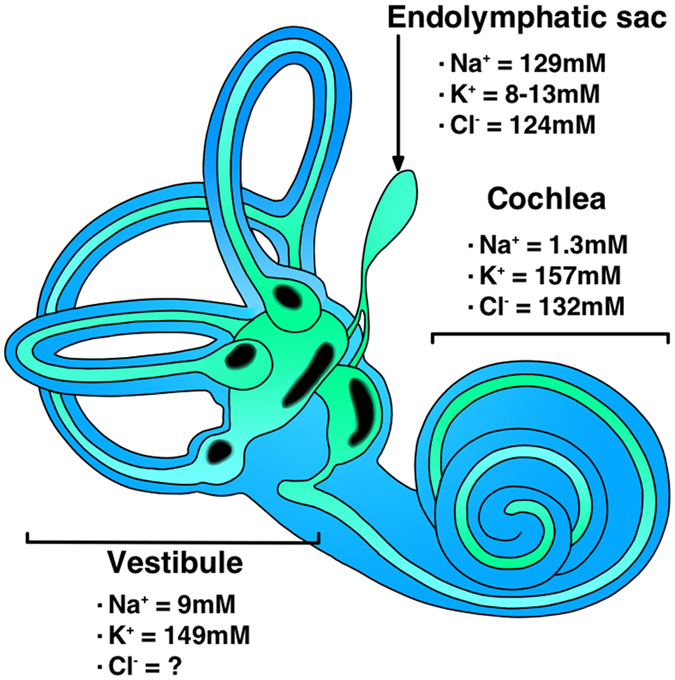
Schematic figure of inner ear structures, and ion composition of the endolymph in each compartment. The black circular areas in the vestibule and cochlear duct contain sensory epithelia.

**Table 1:** Primers used for RT-PCR.

Gene	GenBank Accession No.		Primer	Amplicon size (bp)
GAPDH	NM_002046.5	Forward	CCCCTTCATTGACCTCAACTAC	418
		Reverse	GAGTCCTTCCACGATACCAAAG	
β_1_ adrenoreceptor	NM_000648.2	Forward	TCGTGTGCACCGTGTGGGCC	265
		Reverse	AGGAAACGGCGCTCGCAGCTGTCG	
β_2_ adrenoreceptor	NM_000024.5	Forward	GCCTGCTGACCAAGAATAAGGCC	329
		Reverse	CCCATCCTGCTCCACCT	
