# Supplementary material for: β1- and β2-adrenergic stimulation-induced electrogenic transport by human endolymphatic sac epithelium and its clinical implications
Source: Sci Rep. 2017 Feb 6;7:42217. doi: 10.1038/srep42217 (PMC5292703; doi:10.1038/srep42217)
Supplement: Supplementary Table S1 [file srep42217-s1.pdf]

β_1- and β_2-adrenergic stimulation-induced electrogenic transport by human endolymphatic sac epithelium and its clinical implications

Bo Gyung Kim[a], Jin Young Kim[b], JinSei Jung[c], In Seok Moon[c], Joo-Heon Yoon[b, c, d*], Jae Young Choi[c, ,d *], Sung Huhn Kim[c, ,d *]

[a]Department of Otorhinolaryngology, Soonchunhyang University College of Medicine, Bucheon, Republic of Korea, 420-767; [b]Research Center for Natural Human Defense System, Brain Korea 21 PLUS Project for Medical Science, Yonsei University College of Medicine; [c]Department of Otorhinolaryngology, Yonsei University College of Medicine; [d]The Airway Mucus Institute, Yonsei University College of Medicine, Seoul, Republic of Korea, 03722

* Joo-Heon Yoon, MD, PhD, Jae Young Choi, MD, PhD, and Sung Huhn Kim MD, PhD, contributed equally to this work as corresponding authors.

Address correspondence to:

Joo-Heon Yoon, MD, PhD, Jae Young Choi, MD, PhD, and Sung Huhn Kim MD, PhD

Department of Otorhinolaryngology, Yonsei University College of Medicine

50 Yonsei-Ro, Seodaemun-gu, Seoul 120-752, South Korea

Tel.: +82-2-2228-3604; Fax: +82-2-393-0580

*E-mail: jhyoon@yuhs.ac (JHY), jychoi@yuhs.ac (JYC) and fledermaus@yuhs.ac (SHK)

Supplementary table 1. Characteristics of patients with Meniere's disease

Patient number	Sex	Age (years)	Lesion side	Lesion side hearing threshold* (dB HL)	Canal paresis (%)**	Mean number of vertigo episodes/month†	Family history of Meniere's disease	Vestibular migraine‡ comorbidity
1	F	55	R	36.3	20	8.0	-	-
2	M	51	L	43.8	12	3.3	-	-
3	M	38	L	40	62.4	2.0	-	-
4	F	68	L	53	50	4.3	-	-
5	F	61	R	90	1	3.3	-	-
6	M	21	R	56.3	9	5.7	-	-
7	M	61	B	70/80 (R/L)	13.2	4.7	-	-
8	M	46	R	53.8	5	1.7	+	-
9	F	59	R	72.5	62	3.3	-	-
10	F	51	B	75/95 (R/L)	35.8	7.3	-	+

F, female; M, male; R, right; L, left; B, bilateral; *, lesion side hearing threshold was calculated as an average of the threshold at four frequencies (0.5, 1, 2, and 4 kHz); **, canal paresis value was calculated in bithermal caloric testing, using Jonkee's formula[30]; †, the

mean number of vertigo episodes/month was calculated as the mean number of vertigo spells/month during the 3 months prior to the surgery; ‡, vestibular migraine was diagnosed according to the diagnostic criteria by the Barany Society and the International Headache Society[31]
